# Runaway multi-allelic copy number variation at the α-defensin locus in African and Asian populations

**DOI:** 10.1038/s41598-020-65675-w

**Published:** 2020-06-04

**Authors:** Timothy Hughes, Lars Hansson, Ibrahim Akkouh, Riad Hajdarevic, Jorunn S. Bringsli, Anja Torsvik, Elin Inderhaug, Vidar M. Steen, Srdjan Djurovic

**Affiliations:** 1 Department of Medical Genetics, Oslo University Hospital, Oslo, Norway; 2 NORMENT, Institute of Clinical Medicine, University of Oslo, Oslo, Norway; 3 Institute of Clinical Medicine, University of Oslo, Oslo, Norway; 4 NORMENT, Department of Clinical Science, University of Bergen, Bergen, Norway; 5 Dr. Einar Martens Research Group for Biological Psychiatry, Department of Medical Genetics, Haukeland University Hospital, Bergen, Norway

**Keywords:** Genetic predisposition to disease, Immunogenetics, Haplotypes, Structural variation

## Abstract

Alpha defensins are anti-microbial peptides of the innate immune system. The defensin A1 and A3 genes are located in a repeat array of variable copy number (the *DEFA1A3* locus) and encode the human neutrophil peptides 1, 2 and 3. The possibility that copy number variation (CNV) may be associated with infection susceptibility and autoimmune pathology motivated the study of *DEFA1A3* CNV across populations. We enhanced two existing methods (one qPCR-based and one sequencing-based) to enable copy number estimation that discriminates between *DEFA1* and *DEFA3* genes. We used these methods to quantify A1/A3 copy number variation in 2504 samples from the 1000 Genomes high-coverage dataset as well as performing FiberFISH assays on selected samples to visualize the haplotypes. These methods produce accurate estimates and show that there are substantial differences between populations. The African population is a clear outlier with a high frequency of the ancestral pure *DEFA1* haplotype, but also harbours exceptionally long haplotypes of 24 copies of both *DEFA1* and *DEFA3*, whilst the East Asian population displays the highest mean level of DEFA3 copy number. Further, our findings demonstrate that qPCR can be an accurate method for CNV estimation and that defensins substantially extend the known range of copy number variation for a human protein-coding gene.

## Introduction

The neutrophil is the most abundant granulocyte in human blood and is a central component of the innate immune system. Its granules are loaded with proteins with potent anti-microbial properties^1^ and the most abundant among these are the three highly similar human neutrophil peptides (HNP1-3) which locate to azurophilic (or primary) granules where they comprise 30–50% of granule protein and 5–7% of total cellular protein^2^. During phagocytosis of a target microorganism, the primary granules fuse with phagocytic vacuoles in which they generate high concentrations of these alpha defensins^1^. Defensin hexamers then form pores in the pathogen’s cell membrane leading to permeabilization and cell death^3,4^.

**Figure 1:**
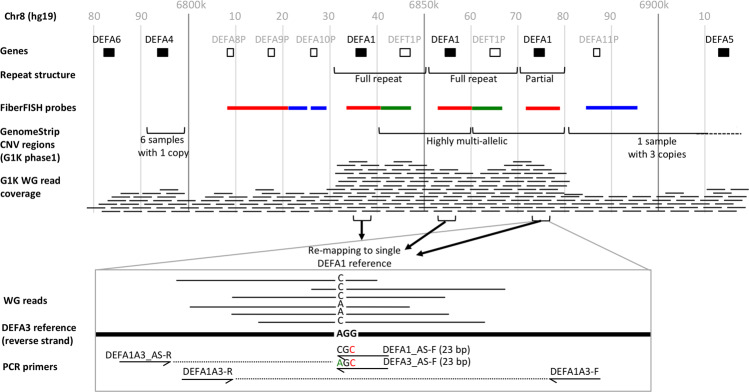
Overview of the defensin-α locus and HTS *DEFA1/DEFA3* ratio estimation. Genes in black and pseudogenes in grey. WG: whole genome sequencing. GenomeStrip CNV regions are the regions defined in Handsaker *et al*.^8^ which do not capture the true nature of the underlying *DEFA1A3* repeat.

In the human genome, the nucleotide sequence for the HNP proteins is found in the alpha defensin gene cluster on chromosome 8 (Fig. 1). *DEFA1-3* (HNP1-3) and *DEFA4* are expressed in neutrophils whilst *DEFA5* and *DEFA6* are expressed in Paneth cells. HNP1 and HNP3 proteins differ from each other at a single amino acid whilst no gene has been discovered for the shorter HNP2. It is therefore thought that HNP2 is a proteolytic product of one or both of HNP1 and HNP3^5^. *DEFA1* and *DEFA3* have long been known to be copy number variant^6^ whilst *DEFA4* is only subject to deletion (and not duplication) in the samples from the 1000 genome (G1K) project (Fig. 1). *DEFA1* has been in a multi-copy array for 25 million years (whilst *DEFA3* is thought to be a human specific variant) and both genes differ in number and location within the array^5^. Thus, the two genes are viewed as variant repeats in gene array haplotypes and the gene locus is designated as *DEFA1A3* by the HUGO Gene Nomenclature Committee.

Most European individuals have 5 or more *DEFA1A3* copies per diploid genome^7^. On a general basis and as one would expect, it has been shown that individuals with high copy number of a gene, express it at a higher level than those with lower copy number^8^. Although this correlation was not observed for *DEFA1A3* in a sample of 111 normal individuals^5^, it is thought that the innate immune system’s need to rapidly produce large quantities of *DEFA1A3* peptides in response to infection probably confers a substantial fitness advantage to individuals with multiple copies of the gene and drives positive selection of gene duplications.

Gene copy number variation (CNV) is a well-known cause of rare genomic disorders, such as several recurrent microdeletions and microduplications that are associated with various neuropsychiatric phenotypes^9,10^. In those cases, the disease mechanism is usually related to the loss or gain of a single copy of the relevant gene(s). Variation in gene copy number can also contribute to more common human diseases such as psoriasis^11^ and asthma^8,12^. Further the relationship between copy number and pathology can be complex, as in the case of the complement C4 gene, where low copy number is associated with susceptibility to systemic lupus erythematosus, whilst high copy number is associated with schizophrenia^13,14^. Some gene-based CNVs show particularly large variation within and between populations, such as the amylase gene *AMY1*, which may be present in 2 to 18 copies per diploid genome^15,16^. In the specific case of *DEFA1A3*, low alpha-defensin gene copy number has been shown to increase the risk of IgA nephropathy and renal dysfunction^17^, whilst increased α-defensins levels have been shown to be a marker for schizophrenia susceptibility^18^ and to be associated with susceptibility to severe sepsis^19,20^.

Interestingly, this type of genetic variation is often overlooked due to the technical challenge of accurate copy number measurement and this has been proposed as one of the possible causes of the missing heritability paradox of many common disorders^21^. Due to the high similarity between *DEFA1* and *DEFA3*, specific measurement of their gene copy number is non-trivial and several different approaches for *DEFA1A3* CNV analysis have been published^5,7,22^.

In the present study, we set out to refine existing methods for the measurement of *DEFA1* and *DEFA3* gene copy number - one based on high throughput sequencing (HTS) and one based on quantitative polymerase chain reaction (qPCR). We then used these methods to generate a comprehensive and accurate overview of *DEFA1A3* copy number variation (including the A1/A3 ratio) in a wide range of human populations. Interestingly, our detailed study of this specific locus also led us to interesting general observations with regards to the estimation and the extent of copy number variation in the human genome.

## Results

### Estimating DEFA1 and DEFA3 copy number using HTS data

We accessed whole genome sequencing data for 2,504 samples of different ethnicities (AFR 661, AMR 347, EAS 504, EUR 503, SAS 489) from the high-coverage phase 3 of the 1000 Genomes (G1K) project and downloaded all reads that mapped to the α-defensin locus (Fig. 1). We estimated *DEFA1A3* copy number with GenomeSTRiP and used the allelic ratio for reads covering the sequence difference between *DEFA1* and *DEFA3* to calculate the gene copy numbers (CN) per diploid genome in each sample. The mean CN across all super-populations is 5.7 for *DEFA1* and 1.4 for *DEFA3* and all super-populations have means approximately equal to these numbers (Table 1). However, these mean statistics conceal important differences in *DEFA1A3* CN distribution between populations, especially regarding *DEFA3*.

**Table 1:** *DEFA1* and *DEFA3* copy number distribution statistics by super-population.

Super-population	DEFA1			DEFA3			DEFA3 Absence (%)
	Min	Max	Mean	Min	Max	Mean	
EUR	0.8	11.6	5.7	0	4.4	1.4	13.1
AFR	0.9	14.5	5.7	0	10.8	1.5	28.7
EAS	0.7	16.1	6.1	0	5.1	1.5	11.5
AMR	0.6	10.3	5.2	0	4.5	1.4	12.7
SAS	1.0	11.1	5.6	0	5.3	1.3	11.2
ALL	0.6	16.1	5.7	0	10.8	1.4	16.3

The DEFA3 absence number is the percent of genotypes in the population with zero copies of DEFA3.

**Figure 2:**
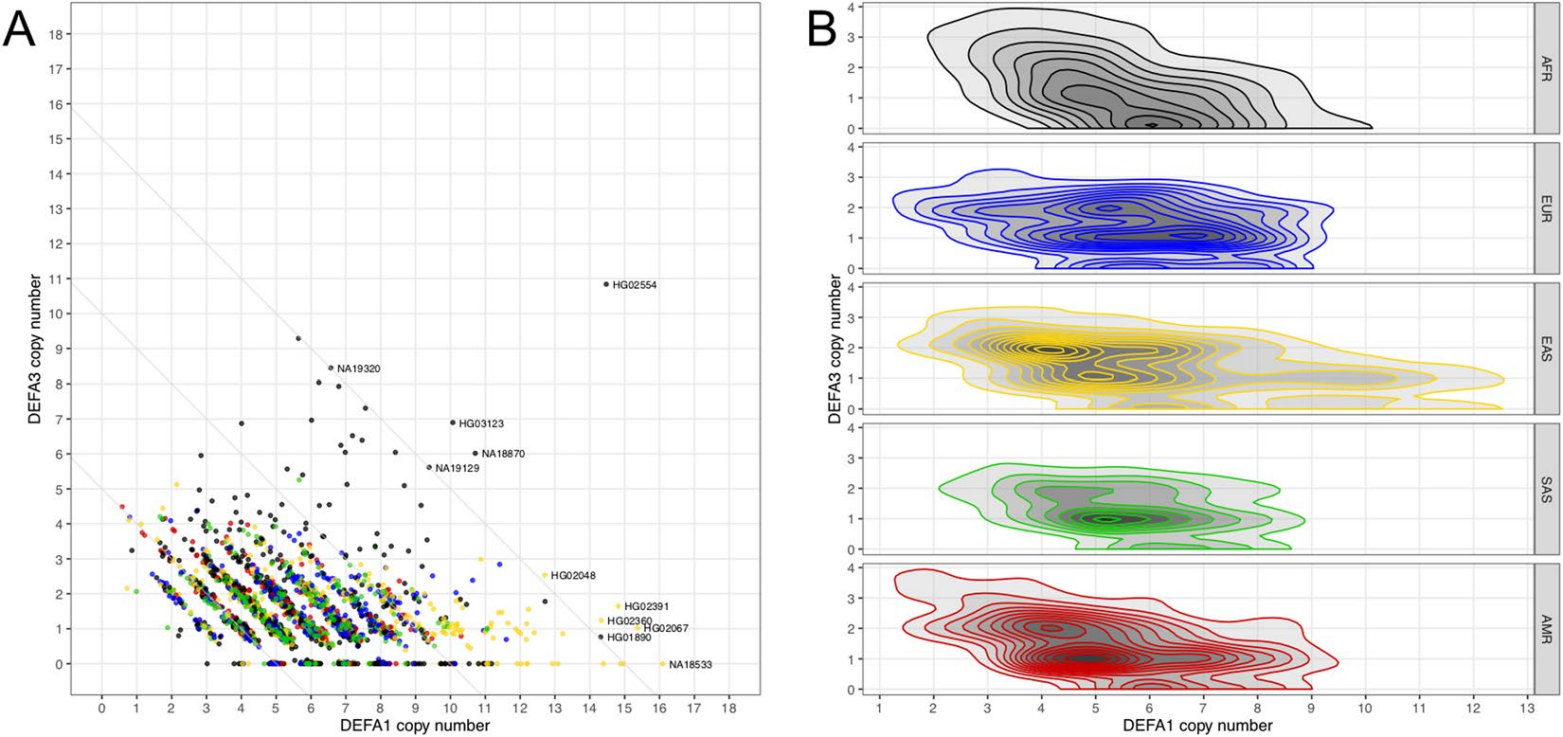
HTS copy number estimates by super-population. (**A**) *DEFA1* and *DEFA3* copy number for all samples (light grey lines delineate genotypes with total CN of 5, 10, and 15). (**B**) 2D densities for each super-population. For both (**A,B**): AFR African (black), EUR European (blue), EAS East Asian (gold), SAS South Asian (green), AMR ad mixed American (red).

First, and most strikingly, individual HG02554 of African-Caribbean ethnicity carries 26 copies of *DEFA1A3* (15 *DEFA1* and 11 *DEFA3*, Fig. 2A). Second, the African super-population differs substantially from the other super-populations in that approximately a third of African samples have genotypes with no *DEFA3* copies (Table 1 and Fig. 2B), but the samples with the highest *DEFA3* copy numbers are also African (Figs. 2A and S2B). We investigated whether the samples with high *DEFA3* CN may be due to unreliable estimates of the *DEFA3* fraction, but this does not appear to be the case (Fig. S3). We also investigated whether the high *DEFA3* CN in Africans may be limited to specific populations, but we found that all 7 African populations have individuals with high *DEFA3* CN (Fig. S4). Third, the Asian super-population has high numbers of samples with more than 10 copies of *DEFA1* (Figs. 2 and S2A). These are primarily Japanese samples (Fig. S5).

We can also use the genotypes to make inferences about haplotype structure. We observe many samples with pure *DEFA1* genotypes with CN ranging from 3 to 16 implying that pure *DEFA1* haplotypes as short as 1 CN and as long as 8 CN exist. We do not observe any samples with a pure *DEFA3* genotype which suggests that pure *DEFA3* haplotypes are either very rare or non-existent (Fig. 2).

### Visualization and characterization of the DEFA1A3 haplotypes

**Figure 3:**
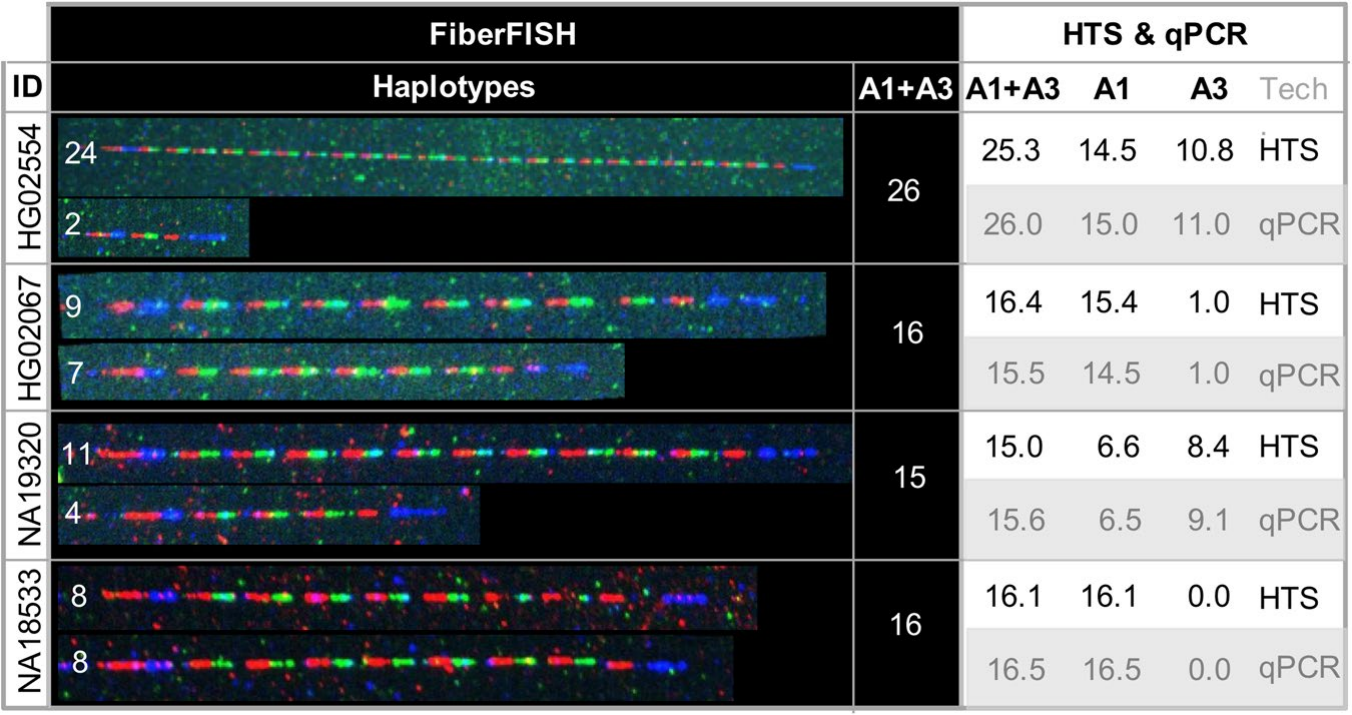
Haplotypes by FiberFISH annotated with *DEFA1A3* copy number. Probe colours as defined in Fig. 1: *DEFA1A3* gene (red), *DEFT1P* pseudogene (green), left flank (red & blue), right flank (blue only). For HG02554, qPCR estimates match FiberFISH estimates exactly as this FiberFISH sample was used for calibration/scaling of the qPCR estimates.

We produced a fully independent validation of our HTS-based *DEFA1A3* CN estimates by performing FiberFISH analysis of four selected samples with high *DEFA1A3* CN values. The fluorescent probes that hybridise to different parts of the alpha-defensin locus (Fig. 1) disclose the haplotype structure in detail (Fig. 3). The genotype of individual HG02554 is highly surprising because its exceptional *DEFA1A3* CN of 26 is not the result of a duplication of the whole repeat array, but rather consists of the extremes of haplotype size with one huge haplotype of 24 CN and one small haplotype of 2 CN (Fig. 3, row 1). It is also evident that genotypes with 15 and 16 *DEFA1A3* CN consist of different combinations of haplotypes: 8 + 8, 9 + 7, and 11 + 4 (Fig. 1, rows 2 to 4).

The HTS data can also be used to shed a broader light on the issue of whole locus duplication by plotting *DEFA1A3* CN vs *DEFT1P* CN for all 2504 samples (Fig. S6). We observe that *DEFA1A3* CN = *DEFT1P* CN + 2, i.e. that there are always two partial repeats (lacking *DEFT1P*) in each genome (Fig. 1). A higher discrepancy would be indicative of more than 2 partial repeats per genome suggesting a duplication of the locus. There is no tendency for samples with high *DEFA1A3* CN to have a greater difference between *DEFA1A3* CN and *DEFT1P* CN suggesting that, as for HG02554, high CN genotypes are the result of *DEFA1A3* haplotypes with a single high CN defensin locus rather than duplication of the whole locus.

### Independent verification of HTS copy number estimates by qPCR

**Figure 4:**
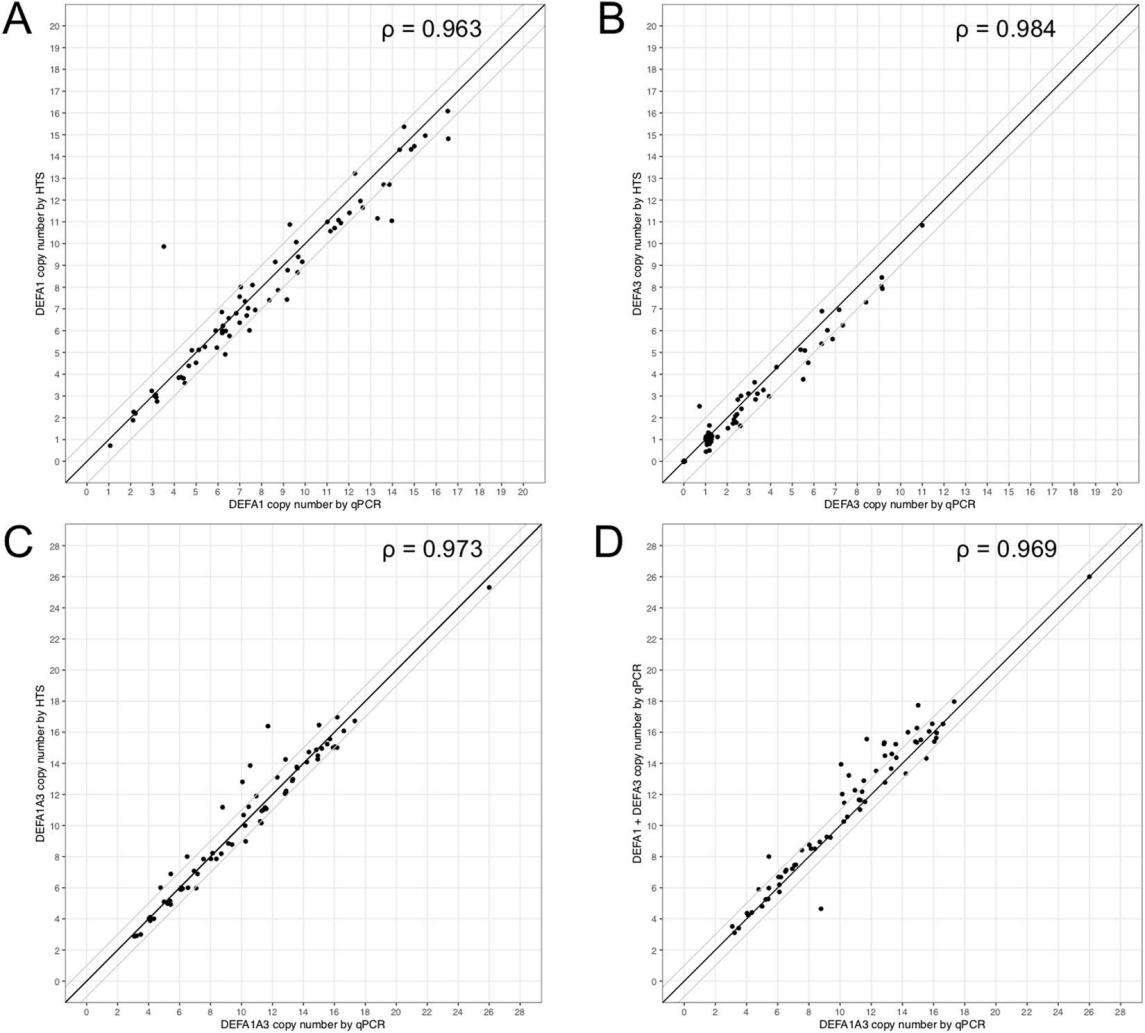
Concordance of HTS and qPCR copy number genotyping methods. (**A**). *DEFA1*, (**B**). *DEFA3*, (**C**). *DEFA1A3*, (**D**). *DEFA1A3* vs *DEFA1* + *DEFA3* (both by qPCR). ρ: Pearson correlation coefficient. Solid line: y = x. Thin lines: y = x + 1 and y = x-1.

The FiberFISH method provides exact quantification of copy number and reveals the haplotypes, but it is an expensive and time-consuming method that cannot be easily applied to many samples and it does not provide an estimate of the *DEFA1/DEFA3* CN ratio. We therefore analysed 69 G1K samples to check the quality of our HTS CN estimates with an orthogonal method (Fig. 4). We generally found a very high correlation between our HTS estimate and the qPCR estimate with all correlations exceeding 96% and the vast majority of estimates deviating from each other by less than 1 CN. The variance of the estimates did not increase as CN increased. Finally, we also checked the consistency of qPCR with itself by comparing *DEFA1A3* CN with the sum of the two separate *DEFA1* CN and *DEFA3* CN assays (Fig. 4D). We found a Pearson correlation of 96.9% and a slight tendency for *DEFA1A3* to generate lower CN estimates than *DEFA1* + *DEFA3*. This slight bias is most likely due to the qPCR scaling factors not being completely exact. We conclude that our HTS CN estimates are accurate in estimating both the total *DEFA1A3* CN and the *DEFA1/A3* CN ratio and thus that they provide an accurate picture of *DEFA1A3* genotype variation across populations.

In the exploratory phase of this work, we tested the qPCR amplifications on the Polymorphism Discovery Resource panel of 24 used by Linzmaier and Ganz^22^, but found that our qPCR *DEFA1A3* CN estimates were substantially and consistently lower than those reported by them (an average discrepancy of 46%) despite the fact that we are using their original primers (Table S5). Given that we find high concordance between our qPCR and HTS estimates (Fig. 4) as well as high concordance between *DEFA1A3* and *DEFA1* + *DEFA3* (both by qPCR), we conclude that the Ganz and Linzmaier primers can be used to provide good estimates of *DEFA1* and *DEFA3* CN, but that the copy numbers reported in their paper are inaccurate^22^. This is probably due to the fact that they do not apply a scaling factor to compensate for the differences in efficiency between target and endogenous control amplifications.

### The DEFA1A3 locus in archaic humans

The HTS data from archaic humans clearly shows that the *DEFA1A3* locus was copy number variant in the Denisova, Altai and Vindija samples (Fig. S8). Further, there is clear evidence of the presence of *DEFA3* variation in the Altai and Vindija samples, but we do not observe the *DEFA3* variation in the Denisova sample (Fig. S9). However, since this is a single sample, it is not possible to conclude whether the *DEFA3* variation was present in Denisova population or not.

## Discussion

### DEFA1A3 copy numbers – Inter-individual variation

Copy number variation has been investigated genome-wide on large numbers of samples by the Wellcome Trust Case Control Consortium^23^ and in phase 1 of the G1K project^8^. Interestingly, in the WTCCC study, the *DEFA1A3* CNV was not scored (perhaps due to strict quality control criteria of CNV typing), whilst in the G1K study, the CN distribution for *DEFA1A3* was estimated to have a mean of approximately 5 (Fig. S7) whereas we estimate it to be 7.1 in the phase 3 data. The reason for this discrepancy is not the difference between phase 1 and 3, but the fact that the automatic method used to identify the basic unit of the CNV incorrectly identifies the partial repeat as part of the basic unit and leaves the left-most part of the full-repeat out (Fig. 1). As a result, these measurements underestimate the true copy number by 2 copies. This specific issue with *DEFA1A3* highlights the importance but also the challenge of correctly identifying the basic unit of a CNV when estimating the copy number of a gene or genomic region. Other CNVs may be affected by a similar issue, in which case, current levels of copy number variation may be underestimated.

Previous work, focused specifically on the CNV at the *DEFA1A3* locus, correctly estimated the mean CN^5,7^ and identified the high fraction of African samples lacking *DEFA3*^24^. However, the difference in distribution between populations and in particular the non-negligible frequency of *DEFA1A3* CN genotypes with 12-16 in African and East Asian populations has not been previously reported (Fig. 2).

Sample HG02554 from an African Caribbean in Barbados with a genotype of 26 CN and haplotypes 24/2 CN (Fig. 3) is an interesting curiosity as it is to our knowledge the longest repeat of a protein coding gene in the human genome. The largest estimated CNV genotype detected genome-wide in the phase 1 of G1K is 15 CN^8^, implying haplotypes that are most likely shorter. Thus, the defensin-α locus and this specific sample demonstrate that the maximal range of copy number variation for protein coding genes in the human genome is substantially greater than previously thought.

### Enhanced DEFA1 and DEFA3 copy number genotyping

A number of techniques have been developed for genotyping *DEFA1A3* copy number. Two methods were published almost simultaneously in 2005. Linzmaier and Ganz proposed using primers that were specific to *DEFA1* and *DEFA3* in qPCR reactions (Fig. 1)^22^, while Aldred, Hollox and Armour used multiplex amplifiable probe hybridization (MAPH) combined with restriction variant digest ratio assays^5^. Following the release of the G1K phase 1 whole genome data, Armour proposed using read coverage to estimate CN^25^, but in its basic form this method does not permit distinction of A1 and A3.

Our results build on and enhance these existing methods. First, we show that Linzmaier and Ganz primers provide good estimates of *DEFA1* and *DEFA3* CN, but that this requires a scaling factor to be applied to compensate for the differences in efficiency between target and endogenous control amplifications. This qPCR method is simpler to perform than the MAPH-based method proposed by Aldred, particularly if one also requires the *DEFA1*/*DEFA3* ratio. Interestingly, the higher mean *DEFA1A3* CN obtained by Linzmaier relative to Aldred in European populations has been remarked upon^7^ and it was suggested that this may reflect limitations of real-time PCR typing for this locus. We find this to not be the case, as long as correct scaling is applied. This is an important conclusion with regards to the use of qPCR in measuring copy number variation. Second, we propose an enhancement to Armour’s HTS-based read coverage estimate, where re-mapping of defensin-α reads to a reference consisting of a single copy of the *DEFA1A3* gene enables accurate estimation of the A1/A3 ratio from the reference and alternative allele depths (Fig. 4). Thus, high throughput methods that enable discrimination between A1 and A3 are now available for analysing the growing number of samples that are whole genome sequenced or for genotyping new samples at the *DEFA1A3* locus specifically.

### Limitations

The primary limitation of this work is that the qPCR method produces a non-integer CN estimate and we observe only mild clustering around integer copy numbers. Further, when we compare qPCR estimates to either HTS estimates or FiberFISH exact copy numbers, although we observe good concordance between methods with little bias (indicating that the methods are accurate), we see that the error margin is approximately 1 CN (Figs. 3 and 4). As a result, we choose to report CN as a continuous variable rather than attempt to estimate an integer copy number. Use of droplet digital PCR would probably have improved the precision of our estimates and made them more amenable to providing an integer CN estimate, but this technology involves higher expense and reduced throughput. Further, it should be noted that the precision of CNV determination at copy number higher than 6 is limited even for droplet digital PCR methods (figure 4.1 in http://www.bio-rad.com/webroot/web/pdf/lsr/literature/Bulletin_6407.pdf and Fig. 3 in^8^).

In this work, we use the FiberFISH copy number to scale our qPCR estimates which may be considered to limit the applicability of the method. However, it should be noted that G1K samples sequenced to very high depth (200X) are publicly available (https://www.illumina.com/platinumgenomes.html) and also provide an accurate scaling factor for the qPCR measurements (unpublished data).

### Evolution of the α-defensin gene locus

*DEFA1* has been present in multiple copies since before the divergence of great apes from gibbons, but chimpanzee, bonobo, gorilla and orangutan all lack *DEFA3*^5^. The whole genome sequence data from the Denisova, Altai and Vindija samples clearly show that these samples are copy number variant at the DEFA1A3 locus (Fig. S8) which was to be expected given the presence of the DEFA1A3 CNV in apes. Interestingly, the same data also show that the *DEFA3* variation was present in the Altai and Vindija samples (Fig. S9). We used our enhanced HTS CN estimation to produce an overview of *DEFA3* frequency across populations. We observe that genotypes lacking *DEFA3* are common in African populations whereas they are markedly less frequent in other populations (Fig. 2). However, genotypes containing *DEFA3* are not rare in African populations and the samples with very high *DEFA3* CN are all African (note that the 24 CN haplotype in HG02554 consists of at least 8 *DEFA3* copies). Further, African samples carrying *DEFA3* are found in all African populations at both low and high CN (Fig. S4). This data does not permit us to draw a firm conclusion on the origin of the *DEFA3* variation. However, in the context of the debate surrounding the geographic origins of modern humans^26–28^, it is intriguing to observe that the *DEFA3* mutation is present in archaic human remains found outside Africa and that modern non-African genotypes mostly contain copies of *DEFA3*, whereas approximately one third of modern African genotypes lack a copy of *DEFA3*.

## Materials and methods

### Copy number estimation using whole genome sequencing data

We downloaded all whole genome sequencing reads mapping to the α-defensin locus (Fig. 1) for the 2504 high-coverage samples (AFR 661, AMR 347, EAS 504, EUR 503, SAS 489) from phase 3 of the 1000 genomes (G1K) project^29^ (http://ftp.1000genomes.ebi.ac.uk/vol1/ftp/data_collections/1000G_2504_high_coverage). We also accessed the pre-computed GenomeSTRiP^8^ metadata for these samples on Google Storage (gs://mccarroll-gs-1000g/md). We estimated DEFA1A3 and DEFT1 copy number for each sample by running GenomeSTRiP (svtoolkit_2.00.1949.tar.gz) with the GSMASKMODE set to MXINC as we found that it was consistent with the results from the default mask mode, but gave more clustering around integer values (full details in the supplementary materials). These are our high-throughput sequencing copy number estimates (HTS CN).

In order to estimate *DEFA3* copy numbers, we first extracted all reads from genomic regions containing *DEFA1A3* and then mapped them to the fasta sequence of the partial repeat (8:6872895-6876338) using the MEM algorithm of BWA^30^. We performed joint variant calling using GATK’s HaplotypeCaller at the SNP site that distinguishes *DEFA1* from *DEFA3*^31^. This is position 6,873,603 in HG19 or position 709 in our reference sequence for the partial repeat. For each sample, we extracted the reference and alternative allele depths from the VCF file and used their ratio as an estimate of the relative copy numbers of *DEFA1* and *DEFA3* (Fig. 1).

### Haplotype determination by FiberFISH

We used FiberFISH (fluorescence *in-situ* hybridisation) to obtain exact quantification of *DEFA1A3* copy numbers (full details in supplementary materials). In brief, we produced a probe design (Figs. 1 and S1) and then generated the FiberFISH probe amplicons with the Qiagen LongRange PCR Kit (QIAGEN, Hilden, Germany) followed by shipment to Genomic Vision (Paris, France) for addition of fluorophores (Table S2). We selected four samples with high HTS CN and obtained their B lymphoblastoid cell lines from the NHGRI Repository at the Coriell Institute of Medical Research (HG02554, HG02067, GM19320, GM18533). We then generated cell plugs that were shipped to Genomic Vision (Paris, France) for molecular combing on vinyl-silane treated coverslips followed by fluorescent probe hybridisation and imaging.

### Copy number estimation by quantitative PCR (qPCR)

69 samples were selected for qPCR verification of the HTS-based CN estimates in such a way that all CN levels were well represented. This meant that almost all available samples with high HTS CN were selected whereas only a small fraction of available samples were selected at low CN (Table S3). DNA samples were obtained from the NHGRI Repository at the Coriell Institute of Medical Research (Camden, NJ, USA).

To determine the copy numbers of the *DEFA1* and *DEFA3* genes, we used the primer sequences proposed by Linzmeier and Ganz^22^. This involved using four sets of primers (Table S4). The *DEFA1* and *DEFA3* genes differ by one nucleotide only, so to discriminate between them, allele-specific primers were used. The last nucleotide of the forward (plus strand) primers covers the differing nucleotide, and the third to last nucleotide contains a mismatch to increase specificity of hybridization further (Fig. 1). The reverse primer was common for both *DEFA1* and *DEFA3*. In addition to the allele-specific primers, a third primer set was also used, that amplifies both *DEFA1* and *DEFA3*. The myeloperoxidase gene, *MPO*, was used as the endogenous control gene and the PCR reactions were run as described in the supplementary materials.

We also obtained the 24 DNA samples that were originally genotyped by Linzmaier and Ganz from the Coriell Institute of Medical Research (Polymorphism Discovery Resource panel of 24 - M24PDR) and performed qPCR as for the 69 G1K samples.

Theoretically, if the efficiencies of the *DEFAx* amplifications and the endogenous control are both 100%, we can use the standard qPCR normalisation methods^32^ to compute the relative amount of *DEFAx* and endogenous control ${2}^{({{\rm{C}}}_{{\rm{t}},{\rm{MPO}}}-{{\rm{C}}}_{{\rm{t}},{\rm{DEFA}}})}$ and this number can be scaled by 2 to obtain the number of *DEFAx* copies per diploid genome. For *DEFA1A3*, this approach is approximately correct. However, for *DEFA1* and *DEFA3*, although we obtain estimates that are proportional to the true copy numbers, the absolute levels are incorrect. We therefore use the exact FiberFISH copy numbers for sample HG02554 to compute scaling factors for each of the primer sets using the following formula:

$${{\rm{FF}}}_{{\rm{DEFAx}}}^{{\rm{HG}}02554}/{2}^{({{\rm{C}}}_{{\rm{t}},{\rm{MPO}}}^{{\rm{HG}}02554}-{{\rm{C}}}_{{\rm{t}},{\rm{DEFAx}}}^{{\rm{HG}}02554})}$$

where the superscript indicates the sample and FF is the copy number determined by FiberFISH (we estimate ${{\rm{FF}}}_{{\rm{DEFA}}1}^{{\rm{HG}}02554}$ and ${{\rm{FF}}}_{{\rm{DEFA}}3}^{{\rm{HG}}02554}$ by using the HTS estimate of the relative copy numbers of *DEFA1* and *DEFA3*). This resulted in the following scaling factors for the different primer pairs: *DEFA1*: 3.319, *DEFA3*: 6.551, *DEFA1A3*: 1.904. Note how the scaling factor for *DEFA1A3* is close to 2 as it should be if the efficiency of the *MPO* and *DEFA1A3* amplifications are both close to 100% whereas the scaling for *DEFA1* and *DEFA3* is much higher, indicating that the efficiency of the *DEFA1* and *DEFA3* amplifications is much lower than that of *MPO* (which was to be expected due to the mismatch at the third to last nucleotide – Fig. 1).

### Neanderthal and Denisovan data

We obtained data on archaic humans by downloading the whole genome sequence data for the Denisova^33^ (http://cdna.eva.mpg.de/denisova/alignments/T_hg19_1000g.bam), Altai^34^ (http://cdna.eva.mpg.de/neandertal/altai/AltaiNeandertal/bam/AltaiNea.hg19_1000g.8.dq.bam), and Vindija^35^ samples (http://cdna.eva.mpg.de/neandertal/Vindija/bam/Vi33.19.chr8.indel_realn.bam) and inspected these in the IGV browser^36^ for presence of DEFA1A3 copy number variation and DEFA3 variation.

## Supplementary information

Supplementary information.

## Data Availability

The raw data that support the findings of this study are openly available in the 1000 Genomes Project at https://www.internationalgenome.org/ and the DNA and cell materials can be obtained from the Coriell Institute for Medical Research. HTS and qPCR data for the 69 samples and the FiberFISH data for the 4 samples has been deposited in dbVar (accession: nstd187).
