# Supplementary material for: Runaway multi-allelic copy number variation at the α-defensin locus in African and Asian populations
Source: Sci Rep. 2020 Jun 4;10:9101. doi: 10.1038/s41598-020-65675-w (PMC7272440; doi:10.1038/s41598-020-65675-w)
Supplement: Supplementary file 1 — Supplementary information. [file 41598_2020_65675_MOESM1_ESM.docx]

**Supplementary materials**

**Runaway multi-allelic copy number variation at the α-defensin locus in African and Asian populations**

Timothy Hughes, Lars Hansson, Ibrahim Akkouh, Riad Hajdarevic, Jorunn S. Bringsli, Anja Torsvik, Elin Inderhaug, Vidar M. Steen, Srdjan Djurovic

**GenomeSTRiP settings**

The command line used to run GenomeSTRiP is:

classpath="${SV_DIR}/lib/SVToolkit.jar:${SV_DIR}/lib/gatk/GenomeAnalysisTK.jar:${SV_DIR}/lib/gatk/Queue.jar"

java -Xmx10g -cp ${classpath} \

org.broadinstitute.gatk.queue.QCommandLine \

-S ${SV_DIR}/qscript/SVGenotyper.q \

-S ${SV_DIR}/qscript/SVQScript.q \

-cp ${classpath} \

-gatk ${SV_DIR}/lib/gatk/GenomeAnalysisTK.jar \

-configFile ${SV_DIR}/conf/genstrip_parameters.txt \

-R $rmdFile \

-runDirectory ${runDir} \

-jobLogDir ${runDir}/logs \

-vcf $vcfFile \

-O ${runDir}/output_file.genotypes.vcf \

-parallelRecords 100 \

-P genotyping.modules:depth \

-md gs://mccarroll-gs-1000g/md/md_batch1/ACB/metadata.zip \

-md gs://mccarroll-gs-1000g/md/md_batch10/FIN/metadata.zip \

-md gs://mccarroll-gs-1000g/md/md_batch11/GBR/metadata.zip \

-md gs://mccarroll-gs-1000g/md/md_batch12/GIH/metadata.zip \

-md gs://mccarroll-gs-1000g/md/md_batch13/GWD/metadata.zip \

-md gs://mccarroll-gs-1000g/md/md_batch14/IBS/metadata.zip \

-md gs://mccarroll-gs-1000g/md/md_batch15/ITU/metadata.zip \

-md gs://mccarroll-gs-1000g/md/md_batch16/JPT/metadata.zip \

-md gs://mccarroll-gs-1000g/md/md_batch17/KHV/metadata.zip \

-md gs://mccarroll-gs-1000g/md/md_batch18/LWK/metadata.zip \

-md gs://mccarroll-gs-1000g/md/md_batch19/MSL/metadata.zip \

-md gs://mccarroll-gs-1000g/md/md_batch2/ASW/metadata.zip \

-md gs://mccarroll-gs-1000g/md/md_batch20/MXL/metadata.zip \

-md gs://mccarroll-gs-1000g/md/md_batch21/PEL/metadata.zip \

-md gs://mccarroll-gs-1000g/md/md_batch22/PJL/metadata.zip \

-md gs://mccarroll-gs-1000g/md/md_batch23/PUR/metadata.zip \

-md gs://mccarroll-gs-1000g/md/md_batch24/STU/metadata.zip \

-md gs://mccarroll-gs-1000g/md/md_batch25/TSI/metadata.zip \

-md gs://mccarroll-gs-1000g/md/md_batch26/YRI/metadata.zip \

-md gs://mccarroll-gs-1000g/md/md_batch3/BEB/metadata.zip \

-md gs://mccarroll-gs-1000g/md/md_batch4/CDX/metadata.zip \

-md gs://mccarroll-gs-1000g/md/md_batch5/CEU/metadata.zip \

-md gs://mccarroll-gs-1000g/md/md_batch6/CHB/metadata.zip \

-md gs://mccarroll-gs-1000g/md/md_batch7/CHS/metadata.zip \

-md gs://mccarroll-gs-1000g/md/md_batch8/CLM/metadata.zip \

-md gs://mccarroll-gs-1000g/md/md_batch9/ESN/metadata.zip \

-run

The VCF file defining the regions to be analyzed has 2 data lines where SEGA_MX defines the three *DEFA1A3* regions and SEGB_MX defines the two *DEFT1* regions (HG38 ref). We use the HG38 reference when running GenomeSTRiP because the pre-computed GenomeSTRiP metadata uses this reference:

chr8 6971894 SEGA_MX N <CNV> . . SVTYPE=CNV;END=6983960;DUPINTERVALS=chr8:6991054-7003065,chr8:7010157-7022159;GSMASKMODE=MXINC

chr8 6983961 SEGB_MX N <CNV> . . SVTYPE=CNV;END=6991053;DUPINTERVALS=chr8:7003066-7010156;GSMASKMODE=MXINC

**FiberFISH**

**Probe amplifications:** Primer pairs were designed to generate amplicons for the probe design (**Table S2**).  There are many sequence similarities between the probe regions, so care was taken to avoid potential off-target priming sites within the probe regions. Further, some probe regions were large and required two probe amplicons. The probe amplicons were generated with the Qiagen LongRange PCR Kit (QIAGEN, Hilden, Germany). Each amplification was setup on ice, and carried out in separate wells in a 96-well plate. 60 ng of a pool of DNA from four different G1K individuals (HG02037, HG02554, NA18533, and NA19320) was used as template in each PCR. This was combined with a master mix consisting of 5 µl LongRange PCR Buffer, 10X, 2.5 µl dNTP mix (10 mM each), 0,4 µM of each primer, 0,4 µl LongRange PCR Enzyme Mix, and RNase-free water to a total volume of 50 µl. A hot-lid thermal cycler was pre-heated to 93°C, the plate loaded and then subjected to an initial denaturation and activation hold at 93°C for 3 min. Then, depending on amplicon, 26-38 cycles of denaturation at 93°C for 15 s, annealing for 30 s, and elongation at 68°C for 7 min was performed. Products were analyzed with the Agilent DNA 7500 Kit on the 2100 Bioanalyzer system to check product length and purity (Agilent Technologies, Santa Clara, CA, USA).


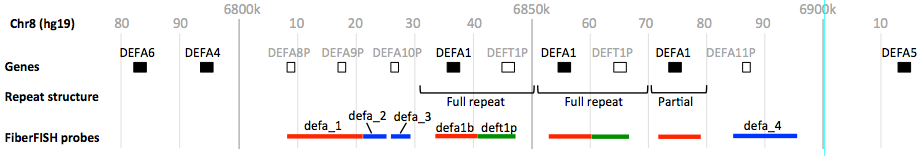


**Figure S1:** Probe design

**Table S2:** Probe amplifications

**Cell plug generation:** The B lymphoblastoid cells were incubated overnight at 37˚C with 5% CO_2_, and then counted (Countess® Automated Cell Counter) and split into two T25 flasks containing 20 ml of RPMI 1640 medium enriched with 15% FBS. Trypan blue assays were performed to obtain cell viability. After sufficient cell growth had occurred, the cells were harvested according to supplier’s instructions (10 minutes at 100 x g at 4C). After harvesting, the cells were resuspended in the appropriate amount of PBS to obtain the desired cell concentration (45µL of cell suspension for one plug) for plug generation according to the manufacturer’s instructions (Genomic Vision, France). Equal volumes of cell suspension and Buffer 2 were added before embedding the solution in DNA plug molds. After 30 min incubation at 4˚C, the generated low-melt-point agarose plugs (~1 million cells per plug, 2 plugs per cell line) were incubated in a water bath (50˚C) overnight in ESP buffer. The next day, enough Buffer 4 was prepared for 3 washing cycles with rotation (old Buffer 4 was replaced with fresh Buffer 4 every hour). The generated plugs were stored in 2ml tubes containing Buffer 5 at 4˚C.

**qPCR**

**Table S3**: Selected samples based on HTS CN for CN verification by qPCR.

**Table S4**: qPCR primers

**PCR reaction details:** The four primer sets were run in duplicate for each sample. Each PCR reaction consisted of two µl 1,25 ng/µl gDNA, 5 µl 2X Power SYBR Green Master Mix (Life Technologies Corporation, Carlsbad, CA, USA), 0,25 µM primers, and nuclease free water to 10 µl final volume. After an initial heating step at 95°C for 10 min to activate the polymerase, 40 PCR cycles were performed. Each cycle consisted of a denaturation step at 95°C for 15 s, followed by a combined annealing and extension step at 63°C for 1 min. A melt curve analysis was performed after each PCR, to assess the specificity of the assay.

**Figure S2:** Density functions for *DEFA1* and *DEFA3* copy number estimated by HTS. AFR African (black), EUR European (blue), EAS East Asian (gold), SAS South Asian (green), AMR ad mixed American (red)

.

**Figure S3:** Reliability of *DEFA1*/*DEFA3* ratio

We compute reliability as log2(total coverage of the SNP distinguishing A1 from A3 / estimated *DEFA1A3* copy number). This plot demonstrates that *DEFA3* CN estimates have a no less reliable estimate of the A1/A3 ratio than samples with lower *DEFA3* CN.

**Figure S4:** Detail of *DEFA1* and *DEFA3* copy number in African populations estimated by HTS.

LWK - Luhya in Webuye (Kenya)

ESN - Esan in Nigeria

YRI - Yoruba in Ibadan (Nigeria)

GWD - Gambian in Western Divisions (Gambia)

MSL - Mende in Sierra Leone

ACB - African Caribbeans in Barbados

ASW - Americans of African Ancestry in SW USA

**Figure S5:** Empirical cumulative distribution function for all populations by super-population: A. *DEFA1* and B. *DEFA3*

**Figure S6:** *DEFA1A3* and *DEFT1P* copy number estimated by HTS

**Table S5:** Copy number estimates for the Polymorphism Discovery Resource panel of 24 - M24PDR. Comparison of our estimates with Linzmaier and Ganz estimates.

**Figure S7:** *DEFA1A3* copy number estimates by GenomeStrip in G1K phase 1

Downloaded (date: 18.12.2018) from: <https://personal.broadinstitute.org/handsake/mcnv_data/variant_report.php?id=CNV_M2_HG19_8_6839961_6859070_8_6859071_6878169>

Location: [chr8:6839961-6859070](http://genome.ucsc.edu/cgi-bin/hgTracks?db=hg19&position=chr8:6838050-6860981) (19Kb), [chr8:6859071-6878169](http://genome.ucsc.edu/cgi-bin/hgTracks?db=hg19&position=chr8:6857162-6880078) (19Kb),


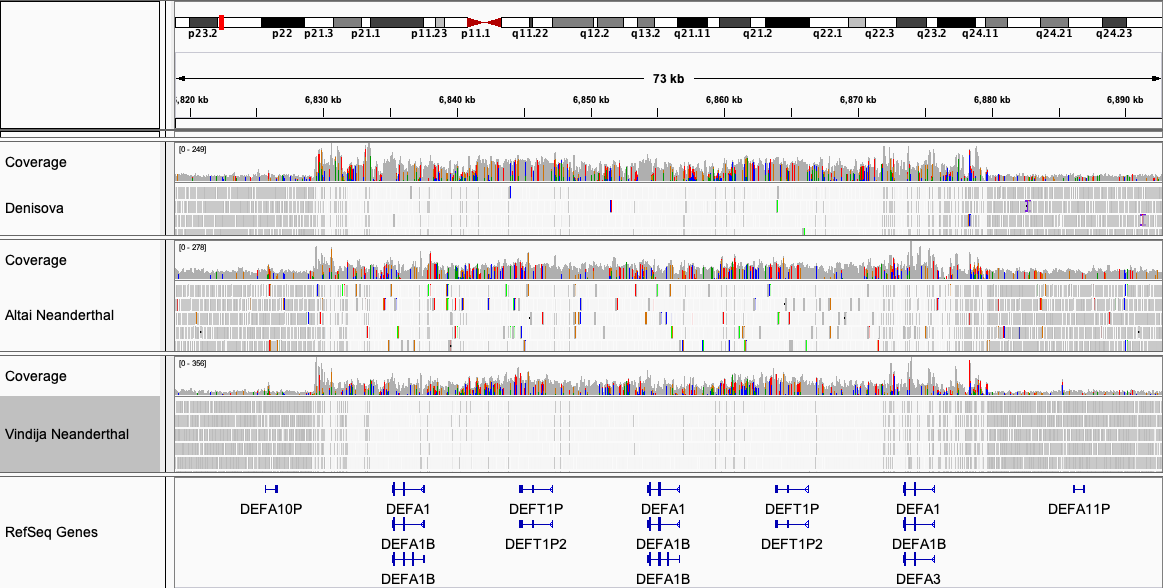


**Figure S8:** IGV display of the *DEFA1A3* locus in HG19. For each of the three archaic human samples, the lower track displays the mapped sequencing reads whilst the upper track summarizes the coverage at each position. Reads are shaded according to their mapping quality, thus the reads in the *DEFA1A3* locus are shaded lighter than the reads in the flanking regions because of the high homology between the *DEFA1A3* repeats in the reference. We observe higher coverage in the *DEFA1A3* locus relative to the flanks in all three samples which indicates the clear presence of copy number variation.


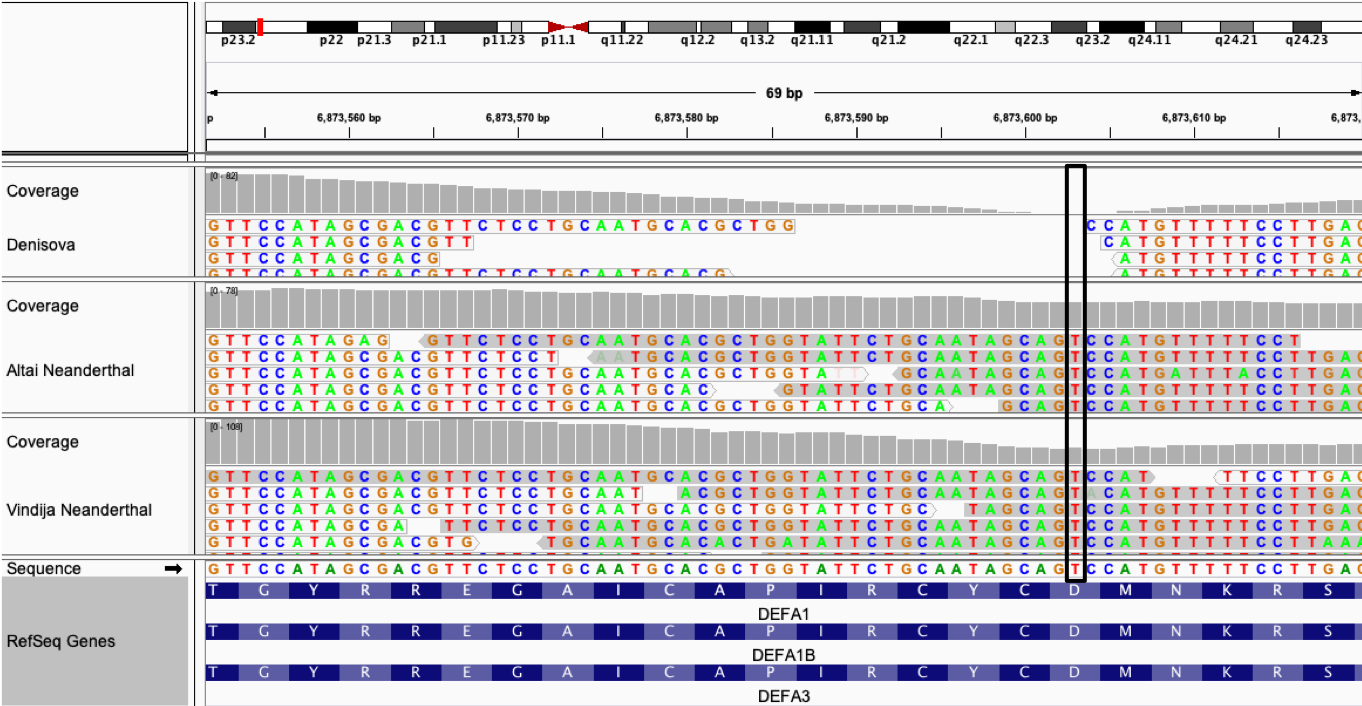


**Figure S9:** An IGV close-up of the *DEFA3* locus in HG19 with Altai and Vindija sequence data showing the clear presence of the *DEFA3* variation at position 6,873,603 (lower two panels), but its absence in the Denisova sample (top panel). The absence of coverage at the *DEFA3* locus in the Denisova sample is due to the fact that the sequencing reads from this sample do not contain the *DEFA3* variant and therefore map better to the two *DEFA1* loci in the reference (to the left in figure S8).
